# A balanced t(5;17) (p15;q22-23) in chondroblastoma: frequency of the re-arrangement and analysis of the candidate genes

**DOI:** 10.1186/1471-2407-9-393

**Published:** 2009-11-10

**Authors:** Salvatore Romeo, Karoly Szuhai, Isao Nishimori, Marije Ijszenga, Pauline Wijers-Koster, Antonie HM Taminiau, Pancras CW Hogendoorn

**Affiliations:** 1 Department of Pathology, Leiden University Medical Center, Leiden, Netherlands; 2 Department of Molecular Cell Biology, Leiden University Medical Center, Leiden, Netherlands; 3 Department of Gastroenterology and Hepatology, Kochi Medial School, Kochi, Japan; 4 Department of Orthopaedic Surgery, Leiden University Medical Center, Leiden, Netherlands; 5 Department of Pathology, Treviso Regional Hospital, Treviso, Italy

## Abstract

**Background:**

Chondroblastoma is a benign cartilaginous tumour of bone that predominantly affects the epiphysis of long bones in young males. No recurrent chromosomal re-arrangements have so far been observed. Methods: We identified an index case with a balanced translocation by Combined Binary Ratio-Fluorescent in situ Hybridisation (COBRA-FISH) karyotyping followed by breakpoint FISH mapping and array-Comparative Genomic Hybridisation (aCGH). Candidate region re-arrangement and candidate gene expression were subsequently investigated by interphase FISH and immunohistochemistry in another 14 cases.

**Results:**

A balanced t(5;17)(p15;q22-23) was identified. In the index case, interphase FISH showed that the translocation was present only in mononucleated cells and was absent in the characteristic multinucleated giant cells. The t(5;17) translocation was not observed in the other cases studied. The breakpoint in 5p15 occurred close to the steroid reductase 5α1 (*SRD5A1*) gene. Expression of the protein was found in all cases tested. Similar expression was found for the sex steroid signalling-related molecules oestrogen receptor alpha and aromatase, while androgen receptors were only found in isolated cells in a few cases. The breakpoint in 17q22-23 was upstream of the carbonic anhydrase × (*CA10*) gene region and possibly involved gene-regulatory elements, which was indicated by the lack of CA10 protein expression in the index case. All other cases showed variable levels of CA10 expression, with low expression in three cases.

**Conclusion:**

We report a novel t(5;17)(p15;q22-23) translocation in chondroblastoma without involvement of any of the two chromosomal regions in other cases studied. Our results indicate that the characteristic multinucleated giant cells in chondroblastoma do not have the same clonal origin as the mononuclear population, as they do not harbour the same translocation. We therefore hypothesise that they might be either reactive or originate from a distinct neoplastic clone, although the occurrence of two distinct clones is unlikely. Impairment of the *CA10 *gene might be pathogenetically relevant, as low expression was found in four cases. Diffuse expression of SRD5A1 and sex steroid signalling-related molecules confirms their role in neoplastic chondrogenesis.

## Background

Chondroblastoma is a benign bone tumour that mainly affects the epiphysis of long bones in young males (male to female ratio 1.5:1; peak of occurrence: second decade) [1-3]. Its nomenclature stems from the presence of cells resembling immature cartilage cells (chondroblasts) set within a distinctive and heterogeneous extracellular matrix [1-3]. The latter is mainly a 'chondroid' extracellular matrix; however, osteoid and fibrous matrix deposits are often observed [1-3]. The lack of a clear-cut, identifiable cartilage extracellular matrix has caused uncertainty on the nature of this tumour [4]. However, recent literature has shown that chondroblastomas share a homogenous expression profile with other cartilage-forming tumours, confirming the cartilaginous nature of this lesion [5,6]. The distinct clinical features of epiphyseal occurrence in pre-pubertal patients suggest a role for growth plate signalling in the pathogenesis of this lesion. Accordingly, we have previously shown IHH/PTHLH and FGF signalling to be active in chondroblastoma [7]. Sex steroids are also likely involved in this process; their role in the pubertal growth spurt and subsequent epiphyseal fusion is well-established [8]. Furthermore, both *in vivo *expression of oestrogen receptors as well as *in vitro *oestrogen/induced proliferation-survival have been previously shown in cartilaginous tumours [9,10]. However, clear understanding of the genetic mechanism driving the pathogenesis of chondroblastoma is lacking. No recurrent chromosomal rearrangements have yet been described (Table 1) [11-15]. Herein, we identified an index case with a balanced translocation t(5;17) with breakpoints mapping close to the *CA10 *and *SRD5A1 *genes and further investigated the involvement of candidate regions/genes in 14 other chondroblastoma cases.

**Table 1:** Conventional cytogenetic findings of chondroblastomas available in the literature. Involvement of chromosome 5 and 17 is shown in bold.

Chromosome changes in chondroblastoma	Reference
46, XX, t(1;1;**5**;**17**;22)(q42;p32;**p13**;**q12**;q21;q12~13)	[9]
46, XY, add(11)(p15)/47, XY, +mar	[10]
46, XY, inv(18)(q11q21)	[10]
47, XY, +der(**5**)t(2;**5**)(q11;**q11**), der(21)t(8;21)(q21q13) 45, XY, del(2)(p23), del(3)(q23q27), dup(8)(q12aq21.3), del(11)(q14q23), 13, add(17)(q25)2	[10]
45, XY, del(2)(p23), del(3)(q23q27), dup(8)(q12aq21.3), del(11)(q14q23), -13, add(**17**)(**q25**)x2	[11]
47, XY, +**5**, t(**5**;**5**)(**p10**;**q10**)	[11]
46, XY, r(4)	[13]
41-46, XY, **-5**, -7, -8, 9qh+c [cp13]	[12]
46, X, -Y, del(2)(q33~35), +7 [cp9]	[12]
46, XY, del(18)(q21) [3]/46XY [8]	[12]
46XY, t(2;22;**5**)(q35;q13;**q31**), inv9(p13q21)c[9]/46, XY, inv9(p13q21)c[11]	[12]
46, XX, der(2)t(2;6)(q31;q25), del(3)(q21~q23), der(6)(6pter->6q25::3q21~q23->3q29::16q23->16qter), der(16)t(2;16)(q31;q23)[19]	[12]
43-47, XX, +8, inv(9)(p12q12~q13)c [cp6]/47-48, XX, +der(1)del(1)(p?)del(1)(q?), +der(2)del(2)(p?), del(2)q?), inv(9)(p12q12~13)c [cp2]	[12]
46, XY, t(**5**;**17**)(**p15**, **q22-23**)	present study

## Methods

### Patients

Paraffin embedded, formalin fixed and, if available, snap frozen tumour samples from fifteen patients were collected. The clinical-demographic details of patients were previously published [5,7]. All samples were handled in a coded fashion, and all procedures were performed according to the ethical guidelines, "Code for Proper Secondary Use of Human Tissue in the Netherlands" (Dutch Federation of Medical Scientific Societies).

### Multicolour fluorescence in situ hybridisation (COBRA-FISH)

For one case (chondroblastoma 13; CB 13), primary cells were isolated from the tumour by using mechanical and enzymatic dissociation procedures. Culture and harvest conditions were performed as described previously [16]. The 43-color FISH staining of every chromosome arm in a different colour combination, digital imaging and analysis were performed as previously described [16]. Hybridisations with individual whole chromosome painting probes labelled with single fluorochromes were used to confirm the detected re-arrangements. Chromosomal breakpoints were assigned by using inverted images counterstained with 4',6-diamidino-2-phenylindole (DAPI; Downers Grove, IL) together with the information derived from the short- and long-arm-specific hybridisation from the COBRA-FISH and FISH mapping data. Karyotypes were described according to ISCN 2005.

### FISH Mapping

Nick translation labelled, large genomic insert clones from the library used for the array-CGH [17] with about 1 Mb spacing were used to map the translocation breakpoints of the involved chromosomes. Consecutive pair-wised hybridisations using distal and proximal probes relative to the estimated breakpoints were selected until a probe with split signal was observed. Based on this, new BAC (Welcome Trust Sanger Institute, Cambridge, UK) and fosmid probes (University of Santa Cruz California, California, USA) covering the 5' and 3' parts of the rearranged regions in CB13 were ordered and then labeled with FITC or Cy3 d-UTPs using a random prime labelling kit (Invitrogen, Breda, The Netherlands) according to the manufacturer's instructions, with slight modifications [18]. Hybridisation was performed on slides from metaphases of CB as previously described [18].

### Array-Comparative Genomic Hybridisation (array-CGH)

The presence of extra copy number changes in CB13 was explored by array-CGH using the 1 Mb large genomic insert clone set distributed by the Wellcome Trust Sanger Institute. DNA was isolated from snap-frozen tissue as previously described [17]. Array printing, hybridisation, and image-acquisition procedures were performed according to a previous report [17].

### Interphase FISH

Four micrometre-thick slides were prepared from snap-frozen tissue from the fifteen cases previously mentioned. Two split-apart probe sets at the CB13 breakpoint regions were selected, set 1: RP11-422F20 and RP11-927L14 (Wellcome Trust Sanger Institute, Cambridge, United Kingdom) on chromosome 5; and set 2: G248P87148D1 and G248P87022G6 on chromosome 17 (University of Santa Cruz California, California, USA). Probes were labelled by standard nick translation with biotin-16-dUTP or digoxygenin-11-dUTP (Roche, Basel, Switzerland). The hybridisation solution contained 50% formamide, 10% dextran sulphate, 50 mM sodium phosphate, pH 7.0, 2 SCC, 3 ng/μl of each probe, and a 50-fold excess of human Cot-1 DNA (BRL-Life Technologies, Rockville, MD). Hybridisation and detection were performed as described previously [19,20]. Slides were analysed by fluorescence microscopy (microscope model DMRXA; Leica Microsystems, Cambridge, UK). Image capture was performed by a monochrome CCD camera (COHU, San Diego, CA) attached to the fluorescence microscope and commercial software (Q-FISH; Leica Imaging Systems). Confocal laser scanning microscope (LSM510, Zeiss) in a multi-track setting was used to combine images from phase contrast with epifluorescence to recognise multinucleated giant cells in CB13. Five *bona fide *multinucleated giant cells were recognised and evaluated. All slides were first evaluated by H&E staining and contained >70% neoplastic cells. Two-hundred random nuclei were scored for detection of aberrations.

### RT-PCR

Possible fusion products deriving from balanced translocation in the index case were explored by RT-PCR. Exons involved in eventual chimera formation were predicted and a pool of primers were designed flanking the involved exons; different combination of these primers are predicted to amplify both the normal product (used as a positive control for the reaction) as well as the eventual fusion products. RNA was extracted and cDNA was synthesised as previously described [5]. PCR reactions were performed in a GeneAmp PCR system 9700 (Applied Biosystems, Foster City, CA) on 1 μL cDNA in a 25 μL reaction containing 10 pmol of each primer, 1.5 μmol/L MgCl2, 1 × PCR buffer II, and 0.5 unit AmpliTaq (Roche). A reaction omitting the DNA template was run as a negative control. PCR-products were analysed by electrophoresis using a 1.5% agarose gel and ethidium bromide staining for visualisation.

### Immunohistochemistry

The levels and patterns of protein expression of steroid-5-alpha-reductase, alpha polypeptide 1 (SRD5A1) and carbonic anhydrase-related protein (CA-RP X the protein product of the *CA10 *gene), two genes respectively located close to the re-arranged regions on chromosome 5 and 17, were determined via immunohistochemistry. The former gene is involved in sex steroid hormone metabolism. Given the specific epidemiology of chondroblastoma (i.e., more often affecting males and occurring in pre-pubertal individuals), we consider it worthy to further explore the sex steroid pathways. Subsequently, the level of expression of oestrogen receptor alpha (ESR1), androgen receptor (AR) and Cytochrome P450, family 19, subfamily A, polypeptide 1 (CYP19A1) were also studied. Immunohistochemistry was performed as previously described [7] with slight modifications: Power Vision plus (Immunologic, Duiven, the Netherlands) was used instead of the SABC method, and visualisation was performed using 3,3'-diaminobenzidine chromogen (DAB plus, Dako SA, Glostrup, Denmark) (details are given in Table 2). The slides were independently evaluated by two pathologists (SR and PCWH). All the tumours were scored using the sum of intensity of signal (possible range: 0 = no expression; 1 = weak expression; 2 = moderate expression; 3 = strong expression) and the number of positive cells (% tumour cells: 0 = 0%; 1 = 1-25%; 2 = 26-50%, 3 = 51-75%; 4 = 76-100%) as described previously [7]. The intracellular (nuclear, cytoplasmic, and membranous) and the intra-tumoural (matrix rich areas or cellular areas) localisation of immunoreactivity were noted. Particular attention was paid to multinucleated giant cells. Given the heterogeneous location in the same tumours, the intensity of signal and the distribution of staining were also evaluated on a six-tiered scale: negative, weak focal, moderate focal, weak diffuse, moderate diffuse, and strong diffuse.

**Table 2:** Details of the antibodies used for IHC.

Antigen	Source	Clone	Staining	Positive control	Dilution	Pre-incubation	Antigen Retrieval
**CA-RP X**	Experimental (22)	MC	Cytoplasm Nuclear	Brain	1:50	Foetal calf serum 20 min	None
**SRD5A1**	Novus Biological	PC	Cytoplasm Nuclear	Prostate Carcinoma	1:100	None	Citrate buffer/10 min mwo
**ESR1**	Zymed	PC	Nuclear	Breast Carcinoma	1:200	Non-Fat Dry Milk 20 min	TrisEDTA/10 min mwo
**AR**	Dako	AR441	Nuclear	Cervix	1:400	none	TrisEDTA/10 min mwo
**CYP19**	Abcam	PC	Cytoplasm	Placenta	1:300	Non-Fat Dry Milk 20 min	Citrate buffer/10 min mwo

MC: monoclonal, PC: polyclonal, Mwo: microwave oven.

Statistical analyses for possible correlation between level of expression of the studied molecules and age and gender of the patients, including Fisher's exact test, Kruskal-Wallis test and Mann-Whitney test as appropriate, were performed with SPSS 11 software package.

### Expression data meta-analysis

We explored significantly differential expression in chondroblastoma versus normal growth plate by interrogating the expression array dataset we previously generated [5,21]. Genes significantly differentially expressed were identified with *limma *(Linear Model for Microarray Analysis) as previously described [5]. Furthermore, a global test for the KEGG pathway androgen and oestrogen metabolism http://cgap.nci.nih.gov/Pathways/Kegg/hsa00150 was performed to identify eventual significant altered expression levels of group of genes involved in this pathway [22].

## Results

**Figure 1:**
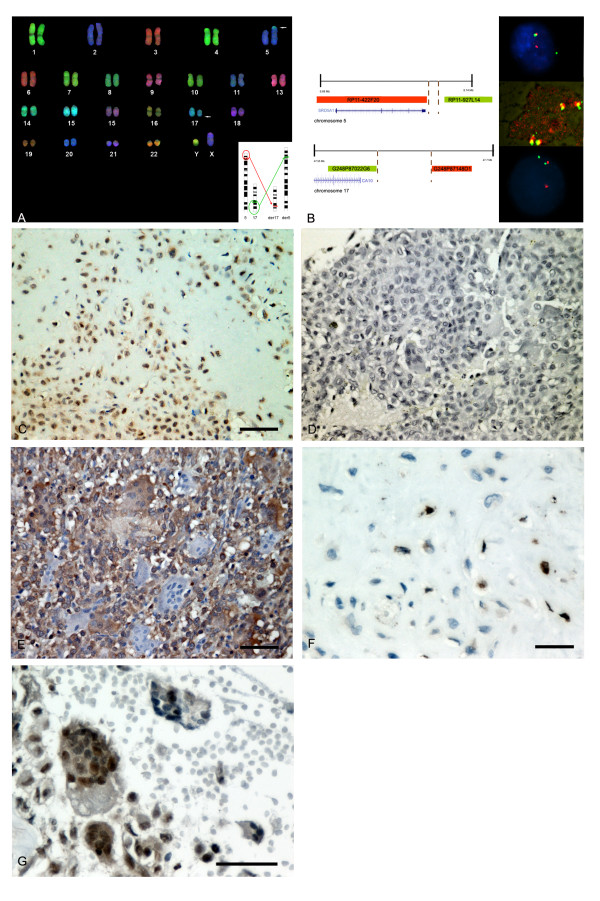
**From identification of an index case to frequency of the re-arrangement and analysis of the candidate genes**: A) COBRA-FISH molecular karyotypes on metaphases from CB13 shows translocation between the short arm of chromosome 5 (white arrows) and the long arm of chromosome 17 (white arrows) (a schematic view of the rearrangement is given in the inset). B) The schematic view recapitulating the results of the FISH mapping (left) (chromosome 5 in the upper part and chromosome 17 in the lower part) The probes that resulted translocated are shown in red, and the retained ones are shown in green. On the right (from top to bottom), interphase FISH shows splitting of the probes covering chromosomes 5 (top) and 17 (bottom) breakpoint regions in the mononuclear cells of CB13, while no rearrangement was found in multinuclear giant cells (middle: confocal microscopy shows a giant cell with 2 nuclei with a normal paired red and green signal). C) Diffuse expression was found by immunohistochemistry for SRD5A1 (picture from CB13). Immunohistochemistry for carbonic anhydrase-related protein X is shown: absent signal in CB13 (D) and diffuse signal of moderate intensity in the other cases (E, CB11); heterogeneous staining was found in multinucleated giant cells. F) Scattered cells in matrix-rich areas were found to express AR (CB13). G) Heterogeneous staining was found in multinucleated giant cells for ESR1 (CB7) (magnification bar = 50 μm).

COBRA-FISH based karyotyping of the index case (CB13) showed 46, XY, t(5;17)(p15;q22-23)[20]/idemx2[5]/46, XY[4] (Figure 1A). The presence of metaphase cells with both rearranged and normal karyograms excluded the possibility of a constitutional translocation. The balanced nature of the rearrangement was supported by array-CGH testing with no additional genomic copy number changes. By FISH mapping, the breakpoint in 5p15 was demonstrated to be proximal to the *SRD5A1 *gene region (Figure 1B). The breakpoint in 17q22-23 was proximal to the *CA10 *gene region with a possible involvement of the gene transcription regulatory element (19) (Figure 1B). RT-PCR using potential chimera primer combinations was negative (data not shown).

Interphase FISH on tissue sections of the index case demonstrated that the translocation only occurred in mononuclear cells, while it was absent in multinucleated giant cells (Figure 1B). Rearrangement of chromosome 5 and 17 was found only in the index case and not in the other investigated chondroblastomas (Figure 1B). One case, CB 10, showed three unsplit signals for both the probes on chromosome 5 as well as on chromosome 17, suggesting either a possible trisomy of the two chromosomes or a triploidy in 2% of the cells (data not shown).

Diffuse expression of the SRD5A1 protein, as identified by immunohistochemistry, was found in the index case as well as in the other chondroblastomas (13/13 tested) (Figure 1C). ESR1 (15/15) and CYP19A1 (15/15) showed a similar pattern. AR showed intense positivity in scattered cells in matrix-rich areas in 5 out of 15 cases (4 male and 1 female) (Figure 1F) including the index case (Table 3). Absent CA-RP X protein expression, as identified by immunohistochemistry, was found in CB13 (Figure 1D), three cases showed low expression and the rest showed weak to moderate intensity in most of the neoplastic cells (Figure 1E). Moderate focal staining was found in multinucleated giant cells for all the antibodies tested (Figure 1G). No significant correlation was found between level of expression of the studied molecules and age and sex of the patients.

**Table 3:** Results of immunohistochemistry

Case number*	Age	sex	Location	SRD5A1	ESR1	AR	CYP19	CA10
CB 2*	30	F	Humerus	3	4	0	4	4
CB 3*	15	M	Humerus	4	7	0	4	4
CB 4*	18	M	Tibia	5	6	4	3	5
CB 5*	14	M	Tibia	6	5	4	6	6
CB 6*	12	F	Humerus	5	7	0	6	3
CB 7*	27	M	os ischium	6	7	0	5	3
CB 8*	11	M	Femur	NA	5	0	0	4
CB 9*	12	M	Femur	4	7	0	3	2
CB 10*	16	M	Femur	6	3	0	5	2
CB 11*	12	M	Tibia	3	3	2	3	3
CB 12*	17	F	Humerus	6	3	0	3	5
CB 13*	**18**	**M**	**Humerus**	**6**	**7**	**4**	**3**	**0**
CB 15*	14	F	Humerus	6	7	3	3	3
CB 16*	18	F	Humerus	6	7	0	2	2
CB 17*	15	M	Humerus	NA	7	0	4	4

Sum score is reported per each case per each antibody tested. M: male, F: female (age is reported in years) *The details of these cases were previously published (4). The index case is shown in bold. NA: not available; CB: Chondroblastoma

The meta-analysis showed that only the gene *BST2 *(CDNA FLJ59809 complete cds, highly similar to Bone marrow stromal antigen 2) (accession number: NM_004335) had significantly higher expression in growth plates versus chondroblastomas (adjusted p = 0.008). Global testing did not show any significant differences in the group of genes present on the expression array that are involved in androgen and oestrogen metabolism.

## Discussion

We report a novel balanced translocation t(5;17) in a chondroblastoma case. No recurrent chromosomal changes have ever previously been described in chondroblastoma (Table 1) [11-15]. Even though involvement of either chromosome 5 or 17 has been described in 5 out of the 13 karyotypes reported thus far, no recurrent chromosome band involvement has been shown (Table 1) [11-15]. FISH mapping identified breakpoints that occurred close to the *SRD5A1 *and *CA10 *genes, which are located on chromosome 5 and 17, respectively. We found no chimera product by reverse transcriptase PCR. Since the FISH mapping identified that the breakpoints occurred outside the coding region, this translocation is hypothesised to interfere with regulatory regions of the nearby genes. In the region of the breakpoints, no other genes are reported.

CA-RP X (Carbonic anhydrase related protein X), the protein product of *CA10*, is a member of the CA family, and SRD5A1 is involved in sex steroid metabolism. The CAs (carbonic anhydrases) are monomeric zinc-metallo enzymes that catalyse the reversible hydration of CO_2_. These enzymes participate in a variety of biologic processes, including calcification and bone resorption. The human CA gene family includes twelve active iso-enzymes that exhibit characteristic catalytic activity (CA I-IV, VA, VB, VI, VII, IX, XII, XIII and XIV) and three isoforms that lack the CA activity due to the absence of one or more of three zinc-binding His residues, which are critical for CA catalytic activity. Thus, the latter have also been designated CA-related proteins VIII, X, and XI. Recent studies have addressed the role of CAs in cancer; notably, CA IX and CA XII have been reported to be over-expressed in several carcinomas [23]. It has been hypothesised that these transmembrane CA iso-enzymes may contribute to the tumour micro-environment by maintaining the extracellular acidic pH [24,25]. CA-RP VIII, which does not have the catalytic CA activity as explained above, has been shown to be predominantly expressed in colorectal cancer cells at the tumour-invasion front [23]. However, the mechanism driving this pattern of expression and its possible role in tumour invasion is largely unknown. CA-RP X expression was previously reported to occur only in neural tissue [25,26]. The promoter region of the *CA10 *gene has been intensively investigated; however, no defined sequence has yet been identified [25]. Immunostaining revealed that CA-RP X protein expression was absent in the neoplastic cells of the index case and expressed at low levels in three other cases. The lack of expression may point to the disruption of a yet unknown control element by the translocation. Furthermore, it may indicate that *CA10 *gene has an allele-specific expression pattern. In the other three cases with low expression, another mechanism diminishing the level of expression is hypothesised to occur. In the remaining cases, a variable expression pattern of the protein was observed. However, a pathogenic role of other members of the CA family cannot be excluded.

Sex steroids accelerate longitudinal growth in puberty [8]. Specifically, upon sexual maturation the level of circulating hormones is markedly increased. This is associated with a dramatic increase in the length growth rate - the so-called pubertal spurt. In both boys and girls, oestrogen is a key determinant for the puberty-associated phenomena related to longitudinal growth and bone quality [8,27]. Specifically in boys, most of the testosterone is converted to oestrogen by CYP19; this enzyme is expressed in and around the growth plate and regulates the local concentration of oestrogen [28]. However, a minor role might be also played by androgens. Specifically, androgen receptors are found in the growth plate [29]. SRD5A1 catalyses the conversion of testosterone into the more potent androgen, dihydrotestosterone. This latter hormone is able to bind androgen receptors, which will translocate to the nucleus thereby activating transcription of the downstream genes. Furthermore, dihydrotestosterone is a non-aromatisable androgen (i.e., it cannot be converted to oestrogen by aromatase). Despite the above-mentioned role of oestrogens in promoting the pubertal spurt, its action on growth plate chondrocyte proliferation rate is not completely clear [8]. A low dosage of circulating oestrogens may stimulate length growth through increased growth plate chondrocyte proliferation [8]. On the other hand, high circulating levels of oestrogens can induce growth plate closure through accelerated bone replacement of hypertrophic chondrocytes. Accordingly, ESR1 is mainly found in growth plate hypertrophic chondrocytes [30]. Remarkably, the diffuse ESR1 expression chondroblastoma closely resembles the expression in hypertrophic chondrocytes. We did not find a casual relationship between the presence of the translocation and changes in the levels of SRD5A1expression. However, diffuse expression of SRD5A1, ESR1 and CYP19 in all the chondroblastomas tested, including the index case, was found. AR showed a scattered expression in few cases, mainly in males.

Taken together, our findings show a great similarity of levels of expression, both at the protein level as well as at the mRNA level, in sex steroid signalling-related molecules between growth plates and chondroblastomas. This supports a possible role for this signalling in promoting chondroblastoma pathogenesis.

Finally, we observed intra-tumoural heterogeneity of the staining pattern of multinucleated giant cells. We previously reported that a similar pattern of expression occurs in multinucleated giant cells of chondroblastomas for other molecules [7]. Some multinucleated giant cells might be of syncytial origin or from impaired duplication of neoplastic cells; however, our present findings lead us to reject this latter hypothesis. In the index case, neoplastic mononuclear cells displayed rearrangement of chromosomes 5 and 17. In contrast, the nuclei from multinucleated giant cells did not display rearrangements in the specific chromosomal regions. Taken together, these results favour a clonal origin of the mononuclear cells of chondroblastoma and suggest that the multinucleated giant cells within the lesions might be reactive. Although we cannot exclude the multinucleated giant cells have a different clonal origin, we consider it more likely that the giant cells are osteoclast-like and recruited through the RANK-ligand, which is present on the surface of the chondroblast-like cells of chondroblastoma [31]. This is in parallel to the recruited nature of giant cells in giant cell tumours of bone, which appear to be blood born as shown by their immunophenotype [32].

## Conclusion

In conclusion, we observed a case with a translocation at t(5;17) with potential involvement of two candidate genes. At the genome level, no disruption of the coding sequences was observed; however, involvement of regulatory elements may have occurred. Although direct correlation was observed only in the index case, a certain type of molecular deficiency caused by the translocation might be important for chondroblastoma pathogenesis. Diffuse expression of SRD5A1 and sex steroid signalling-related molecules confirms the role of this pathway in cartilaginous tumours. Additionally, we determined that multinucleated giant cells in chondroblastoma are most likely reactive.

## Competing interests

The authors declare that they have no competing interests.

## Authors' contributions

SR: experimental design, carried out immunohistochemistry, evaluation of the immunohistochemistry and of the interphase FISH, drafting of the manuscript; KS: experimental design, evaluation of the COBRA and metaphase FISH, commenting on the final manuscript; IN: provided the antibody for CA10 and participated in drafting the discussion; MI: carried out optimising and running COBRA and Metaphase FISH and RT PCR; PW: carried out optimising and running interphase FISH; AHMT: participated in acquisition and analysis of data; PCWH: supervision of the experiments, evaluation of the immunohistochemistry, drafting and editing of the manuscript. All authors have read and given final approval of the version to be published.

## Pre-publication history

The pre-publication history for this paper can be accessed here:

http://www.biomedcentral.com/1471-2407/9/393/prepub
